# Potential Mechanisms of Cancer-Related Hypercoagulability

**DOI:** 10.3390/cancers12030566

**Published:** 2020-02-29

**Authors:** Nicola J. Nasser, Jana Fox, Abed Agbarya

**Affiliations:** 1 Department of Radiation Oncology, Montefiore Medical Center, Albert Einstein College of Medicine, Bronx, New York, NY 10467, USA; jfox@montefiore.org; 2 Institute of Oncology, Bnai Zion Medical Center, Haifa 31048, Israel; abed.agbarya@b-zion.org.il

**Keywords:** cancer, thrombosis, endogenous heparin, heparanase, heparan sulfate

## Abstract

The association between cancer and thrombosis has been known for over a century and a half. However, the mechanisms that underlie this correlation are not fully characterized. Hypercoagulability in cancer patients can be classified into two main categories: Type I and Type II. Type I occurs when the balance of endogenous heparin production and degradation is disturbed, with increased degradation of endogenous heparin by tumor-secreted heparanase. Type II hypercoagulability includes all the other etiologies, with factors related to the patient, the tumor, and/or the treatment. Patients with poor performance status are at higher risk of venous thromboembolism (VTE). Tumors can result in VTE through direct pressure on blood vessels, resulting in stasis. Several medications for cancer are correlated with a high risk of thrombosis. These include hormonal therapy (e.g., tamoxifen), chemotherapy (e.g., cisplatin, thalidomide and asparaginase), molecular targeted therapy (e.g., lenvatinib, osimertinib), and anti-angiogenesis monoclonal antibodies (e.g., bevacizumab and ramucirumab).

## 1. Introduction

**Figure 1:**
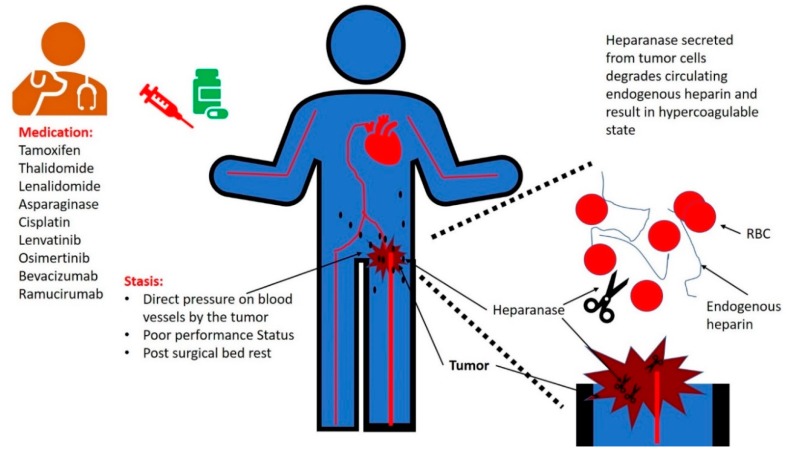
Cancer-associated thrombosis can result from: (1) stasis, i.e., direct pressure on blood vessels by the tumor mass, poor performance status, and bed rest following surgical procedures; (2) iatrogenic, due to treatment with antineoplastic medications; and (3) secretion of heparanase from malignant tumors that results in degradation of endogenous heparin.

Since Trousseau described the correlation between cancer and thrombosis in 1867 [1], there has been no consensus regarding the etiology connecting the two. Virchow’s triad of factors that contribute to thrombosis includes hemodynamic changes, endothelial injury/dysfunction, and alterations in the constituents of the blood [2]. Stasis due to pressure of tumor on venous vessels results in alterations in blood flow and endothelial injury. Poor performance status of patients with cancer has been correlated with a higher risk of thrombosis (Figure 1). This may be due to stasis as well as an indication of the aggressive nature of the malignancy in these patients that results in degradation of their functional capabilities. Several systemic therapies of cancer are correlated with thrombosis, including venous thromboembolism (VTE) and arterial thrombosis. Surgical interventions are also known to be associated with an increased risk of VTE.

Procoagulant molecules secreted from tumor cells are the main cause of hypercoagulability in cancer patients. Heparanase is a mammalian endoglycosidase that degrades heparan sulfate (HS) at the cell surface and in the extracellular matrix. Heparanase is physiologically expressed in platelets and the placenta and is pathologically overexpressed in most malignant tumors. We have shown that heparanase is able to degrade heparin and low-molecular-weight heparin (LMWH) [3]. Transgenic mice overexpressing heparanase in all their tissues have shorter activated partial thromboplastin time (APTT) compared to control mice [3]. We found that a substantial proportion of cancer patients suffering from VTE and treated with standard LMWH doses had subtherapeutic anti-Xa activity [4]. Heparanase overexpression and secretion by cancer cells results in degradation of endogenous heparin and hypercoagulability. Heparanase was also found to induce tissue factor expression in vascular endothelial and cancer cells [5] and to induce dissociation of tissue factor pathway inhibitor from the vascular surface [6]. 

We propose stratifying cancer-related hypercoagulability into two main types. Type I hypercoagulability results from the degradation of endogenous heparin by tumor-secreted heparanase. Type II hypercoagulability includes all the other etiologies, with factors related to the patient, the tumor, and/or the treatment. We will initially review the classical mechanism of cancer-associated thrombosis (type II), and then we will focus on the role of degradation of endogenous heparin by tumor-secreted heparanase (type I).

## 2. Stasis

Direct pressure on blood vessels by a tumor mass can lead to the narrowing of the vessels and subsequent stasis. This results in increased hemodynamic forces on endothelial cells and may induce endothelial dysfunction, thus contributing to the development of vascular pathologies that result in thrombosis [7]. Animal studies on dogs have shown that blood stasis due to an occluded segment of vena cava and/or aorta resulted in blood clot formation within just a few minutes [8,9]. 

Poor performance status is associated with an increased risk for hypercoagulability. White et al. found that one-third of patients with advanced cancer admitted to specialist palliative care units had a femoral deep vein thrombosis (DVT) [10]. Metcalf et al. [11] studied thrombosis risk in ovarian cancer patients. Patients with thrombosis were found to have a worse performance status, with Eastern Cooperative Oncology Group (ECOG) performance status ≥2 in 29.9% of these patients, compared to 9.5% in patients without thrombosis [11]. A prospective study performed bilateral venous Doppler sonography examination of the lower extremities for 44 nonambulatory cancer patients asymptomatic for lower extremity DVT [12]. Asymptomatic DVT was detected in 34% of the patients. DVT was found in 17.4% of patients with one metastatic site and in 52.3% of patients with two or more sites (*p* < 0.01) [12].

Surgical interventions increase the risk of DVT in general, and in patients with malignancies in particular. A high incidence of 26% DVT was found in the perioperative period of neurosurgical patients, with a preoperative diagnosis of DVT in 11% and postoperatively in 15% of the patients [13]. Chaichana et al. [14] reported the incidence of DVT and pulmonary embolism in adult patients undergoing craniotomy for brain tumors. Poor performance status was associated with thrombosis with an odds ratio of 1.04, *p* value < 0.0001 [14]. Older age, preoperative motor deficit, high-grade glioma, and hypertension were also independently associated with increased risk of developing perioperative VTE [14]. Osaki et al. studied the risk and incidence of perioperative DVT in patients undergoing gastric cancer surgery. The preoperative and postoperative incidences of DVT were 4.4% and 7.2%, respectively [15].

## 3. Anti-Neoplastic Medications Associated with Increased Risk of Thrombosis 

Multiple cancer therapies are associated with an increased risk of thrombosis. We do not aim to cover all the medications that are associated with increased risk of thrombosis in this report but will highlight some of the more commonly used agents.

### 3.1. Tamoxifen 

The National Surgical Adjuvant Breast and Bowel Project (NSABP), Protocol B-14, was a double-blind, placebo-controlled trial comparing the effectiveness of tamoxifen in patients with breast cancer, histologically negative axillary nodes, and estrogen-receptor positive. Thromboembolism occurred in 0.9% of patients receiving tamoxifen compared to 0.15% of those receiving placebo [16]. 

Thromboembolic events are more often observed when chemotherapy is given in conjunction with tamoxifen than when tamoxifen is administered alone. The NSABP B-20 study compared chemotherapy plus tamoxifen versus tamoxifen alone in the treatment of patients with axillary lymph node negative, estrogen-receptor-positive breast cancer. VTE was observed in 1.8% of patients treated with tamoxifen alone, compared to 6.5% in patients treated with cyclophosphamide, methotrexate, fluorouracil, and tamoxifen [17]. This increased risk of VTE when tamoxifen is combined with chemotherapy is the main reason that tamoxifen is withheld until chemotherapy treatment is completed.

### 3.2. Chemotherapy 

Multiple studies have shown correlations between chemotherapy and increased incidence of VTE. A retrospective analysis including 17,284 cancer patients found that VTE occurred in 12.6% of the cancer cohort over 12 months after the initiation of chemotherapy, versus 1.4% of controls [18].

(a) Cisplatin. Cisplatin is associated with an increased risk of VTE and arterial thrombosis. A retrospective analysis from the Memorial Sloan Kettering Cancer Center found that 18.1% of cancer patients developed thrombosis during cisplatin treatment. Most of these cases (88%) occurred during the first 100 days from the initiation of cisplatin [19]. A meta-analysis of randomized controlled trials evaluating the incidence and risk of VTE associated with cisplatin-based chemotherapy showed a significantly increased risk of VTE with a relative risk of 1.67 [20]. VTE rates were 1.92% versus 0.79% in patients treated with cisplatin-based and non-cisplatin-based chemotherapy regimens, respectively [20]. A report from the UK National Cancer Research Institute of a randomized trial of patients with advanced gastroesophageal cancer randomized to epirubicin/(fluorouracil or capecitabine) and cisplatin or oxaliplatin found fewer thrombotic events in the oxaliplatin compared with the cisplatin groups, 7.6% vs. 15.1%, respectively; *p* = 0.0003 [21].
(b) Thalidomide. Thalidomide inhibits the production of interleukin (IL)-6, while suppressing proliferation and activating apoptosis of myeloma cells [22]. A study that treated patients with multiple myeloma using thalidomide and dexamethasone in preparation for autologous stem cell transplantation found VTE in 13% and 26% of patients treated with or without low-dose prophylactic warfarin, respectively [23]. A phase III clinical trial of thalidomide plus dexamethasone compared with dexamethasone alone in newly diagnosed multiple myeloma showed that VTE occurred in 19.6% and 2.9% of patients treated with and without thalidomide, respectively [24].
(c) Asparaginase. Asparaginase is an enzyme that degrades L-asparagine, resulting in inhibition of protein synthesis in tumor cells [25]. A retrospective study reported thrombotic complications in adult patients with acute lymphoblastic leukemia receiving L-asparaginase during induction therapy in 4.2% of the patients [26]. A meta-analysis of 1752 patients from 17 prospective trials involving treatment with asparaginase demonstrated a rate of symptomatic thrombosis of 5.2% [27]. The UK ALL 2003 study reported asparaginase-related venous thrombosis in 3.2% of 1824 treated patients [28]. The use of prednisone and asparaginase concomitantly administered in a leukemic patient suffering from a prothrombotic risk factor (such as protein C deficiency, protein S deficiency, antithrombin deficiency, or factor V Leiden) was responsible for the onset of venous thrombosis in the majority of cases [29].

### 3.3. Molecular Targeted Therapies

(d) Lenvatinib is an oral medication that inhibits multiple receptor tyrosine kinases, including vascular endothelial growth factor receptors, fibroblast growth factor receptors, and platelet-derived growth factor receptor alpha [30]. A phase 2 trial treating patients with advanced, radioiodine-refractory thyroid cancer with lenvatinib, reported pulmonary embolism in 3% of the patients and DVT in 3% of the patients [31].
(e) Osimertinib is an epidermal growth factor receptor inhibitor that is implicated with an enhanced risk of thrombosis. The dose escalation study showed that pulmonary embolism occurred in 2.4% of the treated patients [32]. Osimertinib-induced VTE after initiation of osimertinib treatment was reported recently by Shiroyama et al. [33].

### 3.4. Anti-angiogenesis Monoclonal Antibodies

(f) Bevacizumab. Bevacizumab is a monoclonal antibody that targets vascular endothelial growth factor (VEGF) in the circulation. The addition of bevacizumab to irinotecan, fluorouracil, and leucovorin resulted in improvement in survival among patients with metastatic colorectal cancer; however, thrombotic events were higher in patients treated with bevacizumab compared to patients treated with chemotherapy alone (19.4% versus 16.2%, respectively, *p* = 0.26) [34]. Analysis of data pooled from five randomized controlled trials found that the combination of bevacizumab and chemotherapy, compared with chemotherapy alone, was associated with an increased risk of arterial thromboembolism with a hazard ratio of 2.0 [35]. A meta-analysis of 20 randomized controlled trials found that the incidence of arterial thrombotic events in patients receiving bevacizumab was 3.3% [36]. This meta-analysis showed the varying risk for arterial thrombotic events with different malignancies treated with bevacizumab, with the highest relative risk of 3.72 for patients with renal cell cancer, and with the relative risk being 1.89 in patients with colorectal cancer treated [36].
(g) Ramucirumab. Ramucirumab is a monoclonal antibody that targets the extracellular domain of VEGF receptor 2, and thus prevents its activation by VEGF [37]. A phase I pharmacologic and biologic study of ramucirumab reported DVT in 5.4% of the patients [37]. A study comparing ramucirumab versus placebo in combination with second-line chemotherapy in patients with metastatic colorectal carcinoma reported a nonsignificant difference in VTE of 8.2% and 6.3% with ramucirumab and placebo, respectively [38].

## 4. Heparin and Heparan Sulphate

The term ‘heparin’ was derived from the Greek word ‘hepar’, or liver, the tissue from which it was first isolated [39]. In 1925, Howell described the role of endogenous heparin for the fluidity of blood [40]. Heparan sulfate is a family of multiple, closely related yet distinct polysaccharide species [41]. Heparin is structurally related to heparan sulfate but has higher N- and O-sulfate contents [41]. Units of N-acetylglucosamine and glucuronic acid form heparan sulphate [42]. Heparan sulfate is ubiquitously expressed on the cell surface and in the extracellular matrix of all mammalian cells [43] and plays multiple roles in cell–cell interactions. Mutations affecting the biosynthesis of heparan sulphate proteoglycans are implicated in multiple diseases including Simpson–Golabi–Behmel syndrome and Ehlers–Danlos syndrome [42]. 

Most commercial heparin production is derived from pig intestine or bovine lungs [44,45]. Multiple studies have shown that heparin and low-molecular-weight heparin (LMWH) appear to prolong survival in patients with cancer [46,47,48,49]. 

## 5. Heparanase

Heparanase is produced as a proenzyme and is activated via proteolytic cleavage by cathepsin L [50]. This proteolytic cleavage results in the production of two subunits of heparanase that heterodimerize to form the active enzyme [51]. Heparanase in its enzymatically active form [50,52] degrades heparan sulfate at the cell surface and in the extracellular matrix [53,54]. We and others have shown that heparanase is capable of degrading heparin [3,55] and low-molecular-weight heparin [3]. We showed that heparanase degradation of heparin and low-molecular-weight heparin results in neutralization of the anticoagulant properties of these molecules [3]. Moreover, we showed that transgenic mice overexpressing heparanase in all their tissues have shorter activated partial thromboplastin time compared to control mice [3], which could be due to degradation of endogenous heparin by heparanase. 

**Figure 2:**
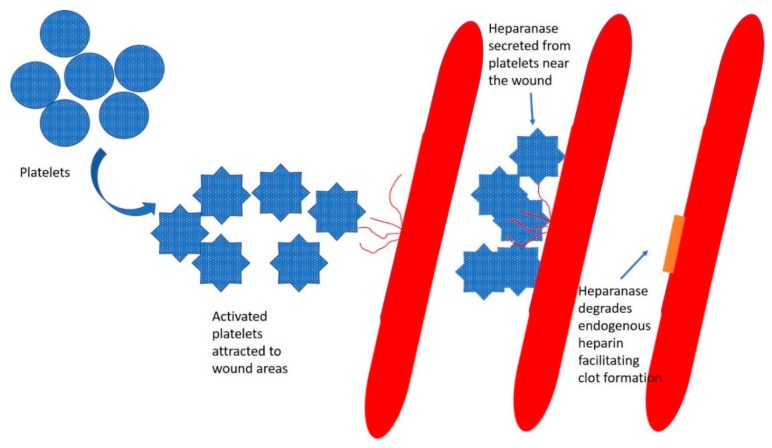
Platelets circulate in the blood. From left to right: Under normal conditions, the platelets are in their nonactive form in the circulation. When an endothelial injury occurs, blood leaks from the injured vessel, and the platelets are recruited to the wound area. The platelets degranulate its content. Heparanase secreted from the platelets degrades endogenous heparin in the blood interface within the wound area to facilitate clot formation. Heparanase also degrades heparan sulphate residues in the injured region as an initial step of wound healing.

Under physiologic conditions, heparanase is expressed mainly in the platelets [56,57,58] and in the placenta [59,60,61,62,63]. Heparanase plays multiple roles in platelets. When an injury occurs, platelets are recruited to the wound region, where they secrete more than 300 active substances from their intracellular granules [64]. One of these molecules is heparanase [55]. The first step in which heparanase is involved is in achieving homeostasis. Heparanase degrades endogenous plasma heparin at the blood–wound microenvironment, facilitating clot formation (Figure 2). The second step is facilitating wound healing. Heparanase degrades the broken heparan sulphate residues at the extracellular matrix of the wounded tissue, clearing the wound area for scar formation and tissue healing. That is followed by the production of a new layer of heparan sulphate covering the healed tissue. Heparanase is expressed at high levels in the placenta [59,61,62,63] and contributes to the high blood vessel density in this critical organ. 

We have cloned multiple splice variants of heparanase [54,65,66,67]; all lack heparanase enzymatic activity. Splice 5 of heparanase, which lacks exon 5, was cloned from human renal cell carcinoma [65]. We cloned heparanase of the subterranean blind mole rat (*Spalax*) and multiple splice variants of it [53,54,66,68] and characterized its heparan sulphate structure [68]. Splice 36 of *Spalax* heparanase functions as a dominant negative to the wild-type enzyme and inhabits heparan sulphate degradation, tumor growth, and metastasis in animal models [54].

## 6. Type I Cancer-Related Hypercoagulability 

Type I cancer-related hypercoagulability is a term that we propose to define thrombotic events in cancer patients that results from lack of sufficient endogenous heparin to maintain the blood in its liquid form. This is mainly due to the degradation of endogenous heparin by tumor-secreted heparanase. Heparanase seems to be the only heparan-sulfate-degrading endoglycosidase [69].

Heparanase was found to be expressed in most malignant tumors. Pancreatic cancers were shown to have heparanase mRNA levels more than 30-fold higher than the levels in normal pancreatic tissues [70]. Kim et al. used in situ hybridization to test for heparanase and found it expressed in 78% of the pancreatic tumors and in none of the normal pancreatic tissues [71]. Heparanase overexpression was associated with angiogenesis and lymphangiogenesis of lung cancer [72]. Cohen et al. showed that heparanase is overexpressed in 75% of lung cancer patients, and its expression correlates inversely with patient survival [73]. A meta-analysis with a total of 27 studies which included 3891 gastric cancer patients showed that higher heparanase expression in gastric cancer is associated with clinicopathologic features of depth of invasion, lymph node metastasis, and TNM stage [74]. All these malignancies are correlated with a high risk of thrombosis, and type I hypercoagulability could be the main reason for that. 

Type I hypercoagulability possibly occurs in other situations, such as in pregnancy [75] where degradation of endogenous heparin by placental heparanase could result in thrombosis. The anticoagulant of choice during pregnancy is low-molecular-weight heparin [75], which is a competitive inhibitor of heparanase [3,4,53]. Patients with multiple traumatic injuries could also be at risk of thrombosis [76]. This increased risk of thrombosis could be due to massive secretion of heparanase from activated platelets and degradation of endogenous heparin. 

## 7. Type II Cancer-Related Hypercoagulability

Type II cancer-related hypercoagulability refers to thrombotic events in cancer patients not related to low endogenous heparin levels. This can occur due to a variety of reasons reviewed here. This includes poor performance status, stasis due to pressure on blood vessels by a tumor mass, or drug-associated thrombosis (Figure 1). 

## 8. Thrombosis as Prognostic Factor in Cancer Patients 

Cancer diagnosed at the same time as or within one year after an episode of VTE is associated with an advanced stage of cancer and a poor prognosis [77]. Patients with cancer have a greater risk both of VTE and bleeding [78]. Early VTE at the beginning of palliative chemotherapy was shown to be a poor prognostic factor in patients with metastatic pancreatic cancer [79]. Patients with pancreatic cancer with early-onset VTE that occurred within 1.5 months after chemotherapy initiation were found to be negative prognosticators for survival outcomes [80]. In patients with cancer and acute VTE, low-molecular-weight heparin was shown to be more effective than an oral coumarin derivative in reducing the risk of recurrent thromboembolism without increasing the risk of bleeding [47,81]. This could be due to the heparanase-inhibiting function of low-molecular-weight heparin [3]. 

## 9. Conclusions and Future Directions

Endogenous heparin is an important component of the balance between blood fluidity and thrombosis. The degradation of endogenous heparin by heparanase seems to play a major role in cancer-associated thrombosis. Developing alternative noninvasive methods to deliver heparin rather than using the subcutaneous or intravenous routes could make this medication more appealing to use in cancer patients, given the introduction of new oral medications [82,83]. Furthermore, developing methods to mobilize endogenous heparin from its reservoirs in the body to the circulation could become the best way to treat cancer-associated thrombosis.
